# Costly Infidelity: Low Lifetime Fitness of Extra-Pair Offspring in a Passerine Bird

**DOI:** 10.1111/evo.12475

**Published:** 2014-07-22

**Authors:** Yu-Hsun Hsu, Julia Schroeder, Isabel Winney, Terry Burke, Shinichi Nakagawa

**Affiliations:** 1 Department of Zoology, University of Otago, Dunedin 9054New Zealand; 3 Evolutionary Biology, Max Planck Institute for Ornithology82319, Seewiesen, Germany; 4 Department of Animal and Plant Sciences, University of Sheffield, Sheffield S10 2TNUnited Kingdom

**Keywords:** Extra-pair paternity, genetic compatibility, good genes, mate choice, multiple mating, polyandry

## Abstract

Extra-pair copulation (EPC) is widespread in socially monogamous species, but its evolutionary benefits remain controversial. Indirect genetic benefit hypotheses postulate that females engage in EPC to produce higher quality extra-pair offspring (EPO) than within-pair offspring (WPO). In contrast, the sexual conflict hypothesis posits that EPC is beneficial to males but not to females. Thus, under the sexual conflict hypothesis, EPO are predicted to be no fitter than WPO. We tested these two hypotheses in a 12-year dataset with complete life-history and pedigree information from an isolated island population of house sparrows (*Passer domesticus*). We compared fitness components of EPO and two types of WPO: (1) WPO from genetically polyandrous “unfaithful” mothers, and (2) WPO from genetically monogamous mothers. We found that all three groups of offspring had similar probabilities of hatching and nestling survival. Unexpectedly, EPO had the lowest probability of recruiting into the breeding population and the lowest lifetime reproductive output. Our results indicate that EPO incurred indirect genetic costs, rather than benefits, which is contrary to indirect benefit models. Importantly, the indirect costs we observed are also underappreciated in current sexual conflict models. Our results call for improved theoretical frameworks that incorporate indirect costs by extending current sexual conflict models.

Social monogamy rarely guarantees genetic monogamy (Westneat and Stewart 2003). Nearly 90% of socially monogamous passerine species are genetically polyandrous (Griffith et al. 2002). Engaging in extra-pair copulation (EPC) is adaptive for socially monogamous males, because males can increase the number of their offspring, thus increasing their direct fitness. These males provide neither resources to extra-pair females nor paternal care to their extra-pair offspring (EPO). Hence, the fitness gain to females that participate in EPC is unclear. EPCs have even been suggested to be maladaptive for females because their social partners may withhold paternal care in response to reduced paternity (Davies et al. 1992; Dixon et al. 1994; Kokko 1999; Arnqvist and Kirkpatrick 2005). Therefore, theoretically, females should only participate in EPC when the benefits balance the costs (Møller 2000; Akçay and Roughgarden 2007). The most frequently suggested benefit of EPCs to females is that EPO are potentially more viable and fertile than within-pair offspring (WPO). These superior EPO will lead to more grand-offspring and, therefore, indirect fitness benefits for females, although EPCs do not guarantee EPO production (Griffith 2007; Sardell et al. 2012).

There are two main hypotheses that posit that female EPC is associated with indirect genetic benefits (Griffith and Immler 2009; Puurtinen et al. 2009). The good genes hypothesis postulates that females will benefit from copulating with high-quality extra-pair males by producing EPO with enhanced genetic viability, assuming that females can infer male genetic quality from male phenotypic traits (Hamilton and Zuk 1982; Hamilton 1990; Houtman 1992). The second hypothesis, the genetic compatibility hypothesis, suggests that females choose extra-pair males on the basis of greater genetic dissimilarity; that is, an extra-pair male has a greater genetic dissimilarity with the focal female than the female has with her social-pair mate (Mitton et al. 1993; Brown 1997). By mating with extra-pair males of greater genetic dissimilarity, females will produce EPO with higher heterozygosity than WPO. This latter hypothesis assumes that the fitness of an offspring is positively correlated with its heterozygosity (Mitton and Grant 1984; Kempenaers 2007). Both hypotheses predict that EPO will have higher fitness than their WPO half-siblings. Further, females pairing with low-quality or genetically similar social-pair males are expected to be more likely to engage in EPC (Gowaty 1996; Whittingham and Dunn 2010). Thus, under these indirect benefit hypotheses, WPO from genetically monogamous mothers that are faithful within a given social pair bond (WPOm; i.e., WPO from mothers who did not produce EPO with a given social partner; Fig.1) are predicted to be fitter than WPO from genetically polyandrous mothers that are unfaithful within a given social pair bond (WPOp; i.e., WPO from mothers who produced EPO with a given social partner, Gowaty 1996). Notably, under this definition, the group “WPOp” includes two classes: (1) WPOp from mixed broods that include both WPO and EPO, and (2) WPOp from broods without EPO but where the mother was unfaithful to the same social male in another breeding attempt (Fig.1). This is because female house sparrows often have multiple breeding attempts, within and between years, with the same partner and each brood may be either WPO-only or a mixed brood (for more details, see Supporting Information S1; Fig.1). Evidence in support of each of the good genes and genetic compatibility hypotheses has been reported in several species (Foerster et al. 2003; Gerlach et al. 2012). However, other studies have provided only limited support for either hypothesis (reviewed in Akçay and Roughgarden 2007).

**Figure 1:**
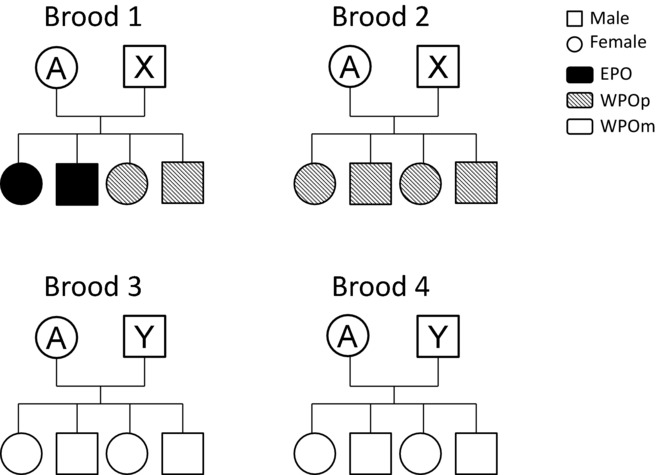
An illustration of the offspring categories used in this study. Circles indicate females and squares indicate males; circles or squares with letters represent adults, those without letters represent offspring. Different letters indicate different adults. Black represents extra-pair offspring (EPO), shaded are within-pair offspring (WPO) from polyandrous mothers who were unfaithful within a given pair bond (WPOp) and white are WPO from monogamous mothers who were faithful within a given pair bond (WPOm). Female A produced four broods in total. These four broods could be between seasons or within one breeding season, and the timing of these broods did not overlap with each other. Female A and male X produced two broods, one of them included EPO, so all the WPO produced by this pair are categorized as WPOp. The same female also produced two broods with another male, Y. Because there are no EPO in broods 3 or brood 4, all the WPO produced by this pair are categorized as WPOm. Note that the descriptions for this illustration are based on the real data in this study.

Such equivocal results may not be surprising because the effect of paternity on fitness is expected to be small under both hypotheses. The underlying assumption of the good genes hypothesis is that the superior genetic quality of extra-pair males results in increased offspring viability (Hamilton 1990; Hasselquist et al. 1996). However, the correlation between offspring viability and paternal phenotypic traits has been shown to be weak, so the difference in fitness between EPO and WPOp is expected to be small and difficult to detect (Møller and Alatalo 1999; Akçay and Roughgarden 2007; Prokop et al. 2012). Similarly, the genetic compatibility hypothesis assumes that females can identify the most compatible males, either by a pre- or postcopulatory choice process (Colegrave et al. 2002; Kempenaers 2007; Leclaire et al. 2012). However, given that females need to consider multiple factors, for example, disease resistance and local or genome-wide heterozygosity, the mechanisms used by females to assess male genetic dissimilarity would need to be complex, and thus difficult to implement with precision (Milinski 2006; Kempenaers 2007). Due to such difficulties, females are often likely to end up mating with genetically suboptimal males when selecting mates based on genetic dissimilarity. Moreover, although positive, the correlation between fitness and heterozygosity is weak (Chapman et al. 2009). Even if highly compatible extra-pair males provide EPO with higher heterozygosity, the increased fitness of EPO from this increased heterozygosity could still be subtle. Therefore, the effect of paternity on fitness under either hypothesis is expected to be small. To detect such effects, we require powerful datasets, such as those provided by long-term studies with large sample sizes and precise fitness measurements.

Potential fitness differences between EPO and WPO may be further obscured because previous studies often targeted fitness components only from specific life-history stages. A more holistic approach may be necessary because the effect of paternity can potentially vary among life-history stages (e.g., Sardell et al. 2011). Therefore, the effects of paternity should be estimated at successive, or even all, life-history stages to understand when the genetic benefits associated with paternity are obtained. To date, studies that provide exact fitness estimates at several life-history stages have been rare, and have so far provided equivocal results (Sardell et al. 2011, 2012; Gerlach et al. 2012; Reid and Sardell 2012).

In contrast to indirect benefits, the sexual conflict hypothesis proposes an alternative explanation as to why females engage in EPC (Parker 1979; Westneat and Stewart 2003). Sexual conflicts occur when males and females have different evolutionary interests (Parker 1979; Arnqvist and Rowe 2005). According to this hypothesis, EPC behavior is only beneficial to males and not to females (Arnqvist and Kirkpatrick 2005). Because extra-pair paternity should be independent of female choice and, thus, male quality, EPO are predicted to be no fitter than WPO (Westneat and Stewart 2003; Forstmeier et al. 2011). This scenario, however, may not be true when chicks from broods with EPO experience fitness costs due to reduced paternal care; such a reduction in paternal care has been observed across numerous bird species (Arnqvist and Kirkpatrick 2005). Because males are not known to be able to distinguish EPO chicks from their own, if males withdraw their care, the detrimental consequence of such a reduction will be experienced brood-wide, that is, both in WPOp and EPO (Kempenaers and Sheldon 1997). In this case, we would expect that WPOp from broods without EPO (i.e., pure broods) would be fitter than offspring (both EPO and WPOp) from mixed broods. However, comparing fitness components between WPOp from pure broods and offspring from mixed broods could be problematic. This is because females producing EPO may benefit from indirect genetic benefits but be disadvantaged by direct costs from male care reduction, if such female benefits and costs occur simultaneously (García-Navas et al. 2013; Griffin et al. 2013). Under this scenario, the fitness of EPO is predicted to be higher than that of WPOp from mixed broods (i.e., maternal half-sibs). Therefore, to assess the effect of male care reduction on offspring fitness, the best solution is to compare the fitness of WPOp from pure broods with that of WPOp from mixed broods (rather than treating offspring from mixed broods as one group).

Here, we present a comprehensive long-term study in which we test the predictions of the indirect genetic benefit and the sexual conflict hypotheses (Table 1; cf. Sardell et al. 2011, 2012; Gerlach et al. 2012; Reid and Sardell 2012). Our study was conducted in a closed population of wild house sparrows (*Passer domesticus*). We have been monitoring this population for more than a decade. The closed nature of this study system with long-term monitoring allowed us to examine how paternity affects: (1) separate fitness components during successive life-history stages: hatching, nestling survival, recruitment, and lifetime reproductive output; and (2) composite fitness, quantified from embryo survival to lifetime reproductive success (LRS). According to both the hypotheses that assume indirect genetic benefits to females, we predict that: (1) EPO will perform on average better than WPOp, both in separate fitness components and in composite fitness, and (2) on average WPOm will fare better than WPOp. Alternatively, under the sexual conflict hypothesis, we expect that (A) offspring from different paternity groups will have the same or similar fitness, or (B) offspring from mixed broods will be less fit than WPOp from pure broods. For prediction (B), we compare WPOp from mixed broods and WPOp from pure broods to avoid the potential complication described above.

**Table 1:** Hypotheses on extra-pair mating in socially monogamous species

Hypothesis		Description	Predicitions of
Benefits hypothesis	Good genes	Females engage in EPC with males of higher quality to obtain “good genes” to produce offspring of higher quality	EPO > WPOp; WPOm > WPOp
	Genetic compatibility	Females mate with extra-pair males of greater genetic dissimilarity to produce offspring with higher heterozygosity	EPO > WPOp; WPOm > WPOp
Sexual conflict	Scenario A	Females engage in EPC because the sets of genes controlling for this behavior are favored in males	EPO = WPOp = WPOm
	Scenario B	Social males reduce paternal care due to the loss of paternity	(EPO + WPOp) in mixed broods < WPOp in pure broods; that is, WPOp in mixed broods < WPOp in pure broods

EPO, extra-pair offspring; WPOp, within-pair offspring from polygamous mothers; WPOm, within-pair offspring from monogamous mothers; pure broods, broods containing only WPO (could be either WPOp or WPOm), mixed broods, broods containing both EPO and WPOp.

## Methods

### Study Population and Data Collection

This study was conducted in the house sparrow population on Lundy Island (51.11N, 4.40W), UK, from 2000 to 2011. Being 19 km offshore, this population is geographically isolated from British mainland populations with only four immigrants in 12 years and three confirmed emigrants (Ockendon et al. 2009; Schroeder et al., unpubl. data).

From 2000 onwards, almost every sparrow in this population has been fitted with a unique color ring combination, along with a numbered metal ring supplied by the British Trust for Ornithology (BTO) for individual identification (Nakagawa et al. 2008; Schroeder et al. 2012). During the breeding season (April to August), we checked nest-boxes and other potential nest sites regularly for active nests. We recorded the first-egg-laying date, the clutch size, and the exact hatch day for each brood. Once the chicks hatched, we visited the nest every two to five days to monitor chick growth and survival until shortly before fledging, that is, 12 days posthatching. We monitored subsequent individual survival through regular re-sightings and recaptures. We used behavioral observations to identify social parentage (Nakagawa et al. 2007). We collected tissue samples from chicks, eggs that failed to hatch, and from adult birds for DNA. Although we did our best to sample every unhatched egg, we could not include all eggs for the hatching analysis because: (1) DNA of some dead embryos was too degraded, (2) no visible embryos existed in some eggs for DNA extraction, and (3) some eggs were removed from nests before collection. We used the genotypes at 13 microsatellite loci to assign genetic parentage and construct a comprehensive pedigree of the population (Dawson et al. 2012; Schroeder et al. 2012).

### Fitness Components

We measured offspring performance at four life-history stages. Our first fitness component was hatching success, defined as whether an egg hatched or not (recorded as hatched = 1, failed to hatch = 0; see below for statistical analysis). Our second fitness component was nestling survival, defined as surviving to day 12 posthatching (survived = 1, failed to survive = 0). Recruitment of each fledgling into the breeding population was the next fitness component, defined as the individual producing at least one egg within the first two years after they reached reproductive maturity (recruited = 1, failed to recruit = 0). We allowed two breeding seasons after a focal individual fledged to estimate recruitment because some individuals did not breed in their first summer. For the analysis of recruitment, we used individuals born up to and including 2009, so that we could assess recruitment up to and including summer 2011. Fourth, for each individual that died before February 2012, we calculated its lifetime reproductive output as the total number of fledglings produced genetically; that is, including both the WPO and EPO that a particular individual produced. Fledglings were defined as the nestlings that survived to day 12 posthatching. We used the number of fledglings instead of eggs because the former is a more precise indicator of lifetime reproductive output than the latter. For the fitness components at successive life-history stages, we used subsets of the data that contained only individuals that survived the preceding life-history stage. This means that we used only individuals that successfully hatched in the analysis of nestling survival, and so on. We also estimated the composite fitness, defined as how many fledglings an embryo identified as EPO, WPOm, or WPOp produced through its lifetime. The composite fitness measurement combined the fitness estimation of embryo survival and the number of fledglings produced by each individual; thus, this fitness estimate can be seen as the overall fitness consequence of a female producing an EPO, WPOm, or WPOp.

### Statistical Analysis

All statistical analyses were conducted in R version 2.13.1 (R Development Core Team 2012). We used the R package MCMCglmm to fit Bayesian generalized liner mixed models (GLMMs), from which we estimated our parameters of interest with Markov chain Monte Carlo (MCMC) methods (Hadfield 2010). This method accounts for overdispersion of count data where necessary (Hadfield 2010). Estimates for fixed effects and their contrasts, for example, the difference between any two paternity groups, are statistically significant when the 95% credible intervals (CIs) of their posterior distributions exclude zero. We reported the means of the posterior distributions and their 95% CIs as parameter estimates from each model. We defined different combinations of inverse Wishart priors for each MCMCglmm model (Table S1).

We conducted two sets of analyses using GLMMs. In the first set, we ran five GLMMs to investigate whether offspring fitness performance is associated with paternity group (EPO, WPOm, and WPOp). Because effects influencing different fitness components vary, we conducted a series of separate GLMMs with different combinations of fixed and random effects for each fitness component. We ran binary GLMMs (binomial error with logit-link function) for hatching success, nestling survival, and recruitment, a Poisson GLMM (Poisson error with log-link function) for lifetime reproductive output, and a zero-inflated Poisson (a combination of a binomial error with logit-link function and a Poisson error with log-link function; Hadfield 2010) for the composite fitness. All GLMMs in this set of analyses are without intercepts. In the second set, we ran four GLMMs to test if WPOp from mixed broods and WPOp from pure broods have different fitness performance for each of the three fitness components: binary GLMMs for nestling survival and recruitment, and a Poisson GLMM for lifetime reproductive output. For more details, see Supporting Information S1.

To test whether the four fitness components at successive life-history stages were associated with paternity within the same pair of social parents, we also conducted another set of analyses to compare the fitness components between EPO and WPOp within the same pair of social parents (using random-slope GLMMs to explicitly compare EPO and WPOp from the same pairs). These GLMMs showed similar results to the results of GLMMs comparing all three groups of offspring (the details of the analysis and results are in Supporting Information S2).

## Results

### Sample Size and the Distribution of EPP

Over 12 years, we collected data from 3285 offspring from 965 broods produced by 436 pairs of social parents. Annually, we collected data on 18–217 broods (Fig. S1). The mean clutch size per brood was 3.6 ± 1.1 (mean ± SD). The full dataset included 591 EPO, 1590 WPOp, and 1104 WPOm (Table S6).

### Fitness Components at Four Life-History Stages

#### Hatching

**Figure 2:**
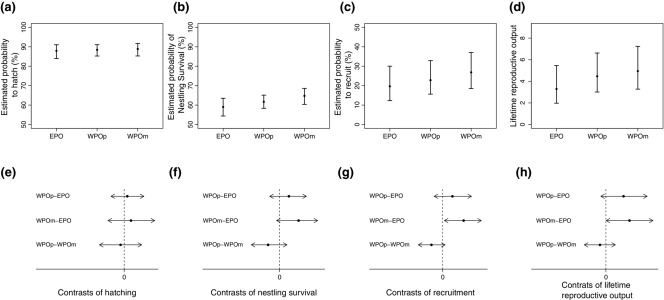
Comparisons of life-history fitness components among offspring from different paternity groups from GLMMs. The first four comparisons are back-transformed estimates of (a) probability to hatch, (b) probability of nestling survival, (c) probability of recruitment, and (d) lifetime reproductive output, defined as the number of fledglings produced by each individual in its lifetime. From (e) to (g) are the pairwise comparisons (on the logit scale) between each two paternity groups of offspring for (e) hatching, (f) nestling survival, and (g) recruitment. The pairwise comparisons (on the log scale) between each two paternity groups of offspring for lifetime reproductive output are presented in (h). EPO, extra-pair offspring; WPOp, within-pair offspring from polygamous mothers; WPOm, within-pair offspring from monogamous mothers.

Hatching success did not differ significantly among paternity groups (Table 2, Fig.2a, 2e). A male offspring was estimated to have a 1% higher chance of hatching than a female offspring, and the difference was statistically significant.

**Table 2:** Results from binomial generalized linear mixed models (GLMMs) with logit-link function, explaining variation in hatching success, nestling survival and recruitment, and a Poisson GLMM, with log-link function, explaining variation in lifetime reproductive output

Model	Hatching		Nestling survival		Recruitment		Lifetime reproductive output	
Estimate	Mean	95% CI	Mean	95% CI	Mean	95% CI	Mean	95% CI
Fixed effects								
EPO	3.37	2.87–3.91	0.68	0.34–1.06	−1.54	−2.12 to −0.94	1.60	0.97–2.19
WPOp	3.45	3.00–3.91	0.89	0.63–1.18	−1.34	−1.84 to −0.81	1.52	1.07–1.94
WPOm	3.54	3.02–4.09	1.14	0.79–1.48	−1.10	−1.63 to −0.61	1.66	1.24–2.08
Sex	0.49	0.18–0.81	−	−	−	−	−0.82	−1.55 to −0.12
WPOp: Sex							0.78	0.01–1.59
WPOm: Sex							0.66	−0.16–1.44
Clutch size	−	−	−0.36	−0.53 to −0.18	−	−	−	−
First-laying day	−	−	0.48	0.26–0.70	−	−	−	−
Random effects								
Biological brood^1^	6.68	4.85–8.82	−	−	−	−	−	−
Growing-up brood^1^	−	−	7.30	5.60–9.13	−	−	0.03	0.00–0.15
Cohort	−	−	−	−	0.61	0.11–1.42	0.29	0.05–0.67
Social parent pair	−	−	−	−	−	−	0.05	0.00–0.20

1 Because we routinely cross-fostered chicks without changing the clutch size during the long-term study on Lundy, for some chicks, the “growing-up” brood identity was different from their biological (original) brood identity.

Rescaled posterior means and 95% credible intervals (95% CIs) under an additive dispersion of 0 are presented. Here “Sex” indicates the difference between male and female, with female as the baseline. EPO, extra-pair offspring; WPOm, within-pair offspring from monogamous mothers; WPOp, within-pair offspring from polygamous mothers.

#### Nestling survival

Among chicks that successfully hatched, paternity group did not influence whether a chick survived to fledging (Table 2, Fig.2b, 2f). An increase of one standard deviation in clutch size (0.9 eggs) was estimated to incur an 8% lower chance of a chick surviving to fledging. Also, an increase of one standard deviation in first-laying day (29.9 days) was estimated to increase nestling survival by 9%. Both clutch size and first-laying day had statistically significant effects on nestling survival (negative and positive, respectively, Table 2).

#### Recruitment

Once a chick survived to day 12 posthatching, EPO were estimated to have the lowest probability of recruiting (20%) into the breeding population. That is, EPO had the lowest chance of producing at least one egg, followed by WPOp (23%) and then by WPOm (27%; Table 2, Fig.2c). EPO were significantly less likely to recruit than WPOm (Fig.2g). There were no significant differences in recruitment between EPO and WPOp and between WPOm and WPOp.

#### Lifetime reproductive output

**Figure 3:**
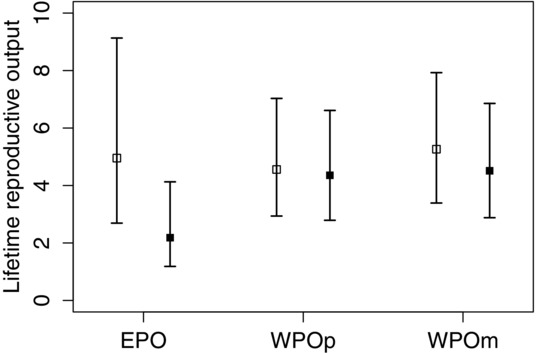
Comparisons of lifetime reproductive output among extra-pair offspring (EPO), within-pair offspring from polygamous mothers (WPOp), and within-pair offspring from monogamous mothers (WPOm). Back-transformed estimates with 95% credible intervals for females (□) and males (▪), separately, from GLMM in each paternity group are presented. Lifetime reproductive output was defined as the number of fledglings that an individual produced through its lifetime.

Recruited EPO were estimated to produce the smallest number of fledglings throughout their lifetime, averaging three. This number was significantly smaller than WPOm (five fledglings), but not significantly smaller than WPOp (four fledglings; Table 2, Fig.2d, 2h). There was no significant difference between the lifetime number of fledglings produced by WPOm and that by WPOp. Male EPO were estimated to produce, on average, two fledglings in their lifetime, which was significantly fewer than other paternity groups, for both males and females, suggesting a sex-specific paternity effect on EPO lifetime reproductive output (four to five fledglings; Table S8, Fig.3).

### Composite Fitness

**Figure 4:**
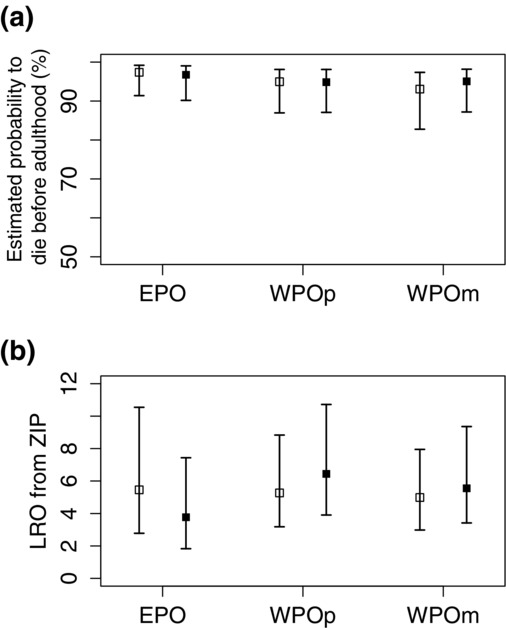
Comparisons of composite fitness among extra-pair offspring (EPO), within-pair offspring from polyandrous mothers (WPOp) and within-pair offspring from monogamous mothers (WPOm) from zero-inflated Poisson GLMM. Back-transformed estimates with 95% credible intervals for females (□) and males (▪), separately, are presented. The composite fitness was defined as (a) for each individual, the probability of failing to survive from embryo to adulthood, which was modeled in the binomial (binary) process; and (b) for surviving adults, the number of fledglings each individual produced in its lifetime (lifetime reproductive output, LRO), which was modeled in the Poisson process.

EPO were estimated to have the lowest composite fitness. The zero-inflated process of the ZIP model showed that EPO had the lowest probability of surviving from embryo to adulthood, which was significantly less than for WPOm but not different from WPOp (Fig.4). However, among individuals that reached adulthood (i.e., the Poisson part of the ZIP model), the paternity group did not influence the total number of fledglings that an individual produced in their lifetime. Neither sex nor the interaction between sex and paternity group influenced composite fitness (Table S9).

### Comparisons between WPOp from Mixed Broods and WPOp from Pure Broods

We included 1414 WPOp in the analyses, among which 607 were born into broods with EPO and 807 into broods without EPO (Table S7). The presence of EPO in brood did not affect the probability of a WPOp fledging or being recruited, or the quantity of its lifetime reproductive output (Table 3).

**Table 3:** Results from the generalized linear mixed models, GLMMs, explaining variation in nestling survival, recruitment, and lifetime reproductive output, for within-pair offspring from polygamous mothers (WPOp) with EPO in broods and WPOp without EPO in broods, using WPOp without EPO in broods as the baseline. Posterior means and 95% credible intervals (95% CIs) are presented

Model	Nestling survival		Recruitment		Lifetime reproductive output	
Estimate	Mean	95% CI	Mean	95% CI	Mean	95% CI
**Fixed effects**						
(Intercept)	2.40	0.43–4.43	−1.58	−2.17 to −1.00	1.35	0.94–1.76
EPO in brood	0.29	−0.24–0.83	−0.47	−0.99–0.06	0.03	−0.35–0.42
First-laying day	0.40	0.08–0.71	−	−	−	−
Clutch size	−0.36	−0.63 to −0.08	−	−	−	−
**Random effects**						
Cohort	14.98	2.27–36.81	0.57	0.00–1.46	0.20	0.00–0.54
Social parent pair	0.24	0.00–0.94	0.18	0.00–0.78	0.05	0.00–0.22
Growing-up brood	6.44	3.97–9.09	0.11	0.00–0.57	0.09	0.00–0.38
Dam identification	0.10	0.00–0.54	0.22	0.00–0.81	0.07	0.00–0.29
**Dispersion**	−	−	−	−	0.60	0.25–0.96

## Discussion

EPO performed similar to or worse than both WPO from monogamous mothers (WPOm) and WPO from polyandrous mothers (WPOp) at every life-history stage we investigated. Over our 12-year study period, EPO were less likely to recruit than WPOm, and produced fewer fledglings than both WPOm and WPOp, although EPO had a similar likelihood of hatching and fledging compared to both WPOm and WPOp. The fitness components of WPOp at these life-history stages were similar to those of WPOm. Moreover, and most importantly, we found that EPO had a lower composite fitness than WPOm and WPOp, providing the strongest evidence that, overall, EPO had a lower fitness than the other groups. Our results do not support either the good genes or the genetic compatibility hypotheses. Furthermore, our results cannot be fully explained by the current sexual conflict models which only considers direct costs to females, because two outcomes are expected under the current sexual conflict models—(A) that the three paternity groups will have similar fitness, or (B) EPO and WPOp from mixed broods will have lower fitness than WPOp from broods without EPO—but our results did not support either of these. This observation, along with our main results, suggests that the reduced fitness of EPO is probably due to indirect genetic costs rather than direct costs.

Our study is not the first to challenge hypotheses positing that females gain indirect genetic benefits from producing EPO. Although some empirical studies support these hypotheses, two meta-analyses of 11 and 10 studies, respectively, detected no difference in fitness measurements between EPO and WPOp at different offspring life-history stages, such as fledging success and recruitment (reviewed in Arnqvist and Kirkpatrick 2005; Akçay and Roughgarden 2007). Recently, using animal models to estimate additive genetic variation, EPO in song sparrows were shown to have lower genetic value to survive to recruitment than the WPOp that the EPO replaced (Reid and Sardell 2012). LRS, however, is a more accurate and complete estimator of fitness than the fitness components included in these meta-analyses. Logistically, obtaining lifelong fitness measurements is extremely difficult, so very few studies have achieved this. So far, only one study of LRS supports indirect genetic benefits of extra-pair paternity: in dark-eyed juncos (*Junco hyemalis*), EPO produced more fledglings than WPO (WPOm and WPOp; Gerlach et al. 2012). However, the other long-term studies that have attempted to quantify LRS reached similar conclusions to the meta-analytic studies. In coal tits (*Periparus ater*), male EPO produced fewer hatchlings throughout their lifetimes compared to male WPOp, but such a difference was not found in female offspring (Schmoll et al. 2009). Similarly, the number of recruited offspring produced by EPO in song sparrows (*Melospiza melodia*) was similar to that of WPOp (Sardell et al. 2012). Our long-term study with a comprehensive pedigree provided complete life-history fitness measurements, yet our results again did not support hypotheses that invoke female indirect benefits.

Furthermore, as mentioned above, our results may not be fully consistent with the scenarios predicted from the current sexual conflict models that only consider direct costs because we found that females unexpectedly suffered indirect costs from low-quality EPO. From an evolutionary point of view, whether EPC behavior can be maintained in a population depends on the tug-of-war between selection favoring EPC behavior in males and selection against it in females (Westneat and Stewart 2003; Arnqvist and Kirkpatrick 2005; Arnqvist and Rowe 2005). To balance these opposing selection pressures on EPC behavior, there are two hypothesized mechanisms: (1) the between-sex genetic correlation hypothesis (Forstmeier et al. 2011) and (2) the male harassment hypothesis (Westneat and Stewart 2003). The between-sex correlation hypothesis posits that EPC behavior in both males and females is controlled by the same set of genes (Halliday and Arnold 1987). Supporting evidence has been reported in captive zebra finches (*Taeniopygia guttata*), in which female infidelity was shown to be genetically correlated with male infidelity (Forstmeier et al. 2011). Additionally, EPC behavior has been shown to be heritable, albeit weakly, in birds (Forstmeier et al. 2011; Reid et al. 2011). Therefore, even though EPC behavior may not benefit a female, she might still express this behavior, given the sets of EPC genes she received from her parents. This is because such sets of genes are positively selected for in males. Such a genetic correlation can be maintained by several mechanisms, for example, pleiotropy or linkage disequilibrium (Halliday and Arnold 1987; Forstmeier et al. 2011). According to the male harassment hypothesis, in species where males force females to engage in EPC behavior, female resistance might lead to higher costs to the female than cooperation because of male aggressive copulation attempts (reviewed in Adler 2010). In this case, females might cooperatively engage in EPC even though such behavior is also costly (reviewed in Westneat and Stewart 2003).

However, as far as we are aware, the current sexual conflict models only consider direct costs (Westneat and Stewart 2003; Arnqvist and Kirkpatrick 2005; Eliassen and Kokko 2008). Therefore, these models cannot explain our finding that EPO attain lower fitness than WPOp and WPOm. One possible explanation for this observed effect of paternity is that extra-pair males may provide low-quality germline “genetic material” to the offspring, even if the “genetic quality” of such males is similar or even higher than that of within-pair males. Extra-pair males may provide low-quality genes through two potential mechanisms—male age and male social status, which are often tightly correlated (Dean et al. 2010). A meta-analytic study on bird populations demonstrated that older males (usually more than two years old) were more likely to gain extra-pair paternity; this pattern is also seen in the Lundy sparrow population, which was also included in the meta-analysis (Cleasby and Nakagawa 2012). An older age might itself indicate that a male has high viability, high foraging ability, and high ability to defend itself from predators and competitors, thus providing direct or indirect benefits to the female (reviewed in Brooks and Kemp 2001; Johnson and Gemmell 2012). However, old males may provide sperm that carry more mutations that have accumulated with age, and other pre- or postmeiotic sperm senescence (Pizzari et al. 2008; Kong et al. 2012), so leading to offspring of poorer genetic quality. EPOs sired by older males might therefore result in EPO having low fitness. The reduced fitness of EPO might also be linked to the social status of extra-pair males. Males with higher social status can potentially attract more females or force more females to engage in EPC (Mennill et al. 2004). Possibly due to a trade-off, in several species male social status has been found to be negatively correlated with male sperm quality (Froman et al. 2002; Rudolfsen et al. 2006; Pizzari et al. 2007). One potential mechanism is that dominant males have more opportunities to mate and produce more sperm in total, while the increased sperm production could lead to more spermatogenesis, and thus higher accumulation of mutations in male germ cells (Miyata et al. 1987; Pizzari et al. 2008; Johnson and Gemmell 2012). Alternatively, males might be able to change their mating strategies, and thus sperm quality, based on their social status (Helfenstein et al. 2010).

Assuming EPO obtained poorer genetic material from the extra-pair male via these two mechanisms, EPO will then be of low quality, resulting in lower LRS for EPO compared to WPO. Moreover, because the distribution of LRS is more skewed in males than females, low-quality males are likely to achieve lower LRS than low-quality females (Fig. S3; Jensen et al. 2004). In other words, due to sexual selection, males have to be of higher quality than females to achieve the same level of reproductive success (Clutton-Brock et al. 1988). This expectation is in accordance with our results, which show that male EPO produced fewer offspring than female EPO. This explanation for a sex-specific effect of paternity on LRS is based on the assumption that EPO are of lower quality (cf. Reid and Sardell 2012). Therefore, further studies are needed to validate this explanation along with other potential reasons for our unexpected finding.

Our results provide strong evidence that female birds suffer indirect costs by producing EPO. Assuming the indirect costs we have found here are widespread, the costs incurred to females via EPO could be a selective force that prevents a population of birds from evolving a high frequency of extra-pair paternity. This mechanism could potentially explain why the frequency of extra-pair paternity is usually low to moderate across species and populations (cf. Griffith et al. 2002). To further investigate the ultimate causes for why females engage in EPC and the consequences of this behavior over an evolutionary time scale, we require new theoretical models extended from the current ones. The current sexual conflict models on why females engage in EPC often consider the production of EPO to be an indirect benefit to females (Kokko 1999; Ihara 2002; Fishman et al. 2003; Arnqvist and Kirkpatrick 2005; Eliassen and Kokko 2008). However, as we reported in this study, producing EPO could incur indirect costs for females. Therefore, existing models for EPC behavior should be extended to include the indirect costs to females.

In conclusion, our comprehensive investigation into the fitness consequences of EPO has shown that females not only fail to gain indirect benefits from EPO, but they also suffer indirect costs by producing EPO. Our results cannot be explained by the currently favored hypotheses based on indirect benefits of EPC. Models based on sexual conflict theory provide the best explanation for our results so far, but the current models have not explicitly considered the possibility of indirect costs to the females. Therefore, these results call for the theoretical expansion of current sexual conflict models to better understand the evolution of EPC behavior.

## Data Archiving

The doi for our data is 10.5061/dryad.25sg7/1.

## Supporting Information

**Supporting Information S1**. Detailed methods for statistical analysis in the main text. **Supporting Information S2**. Models comparing extra-pair offspring and within-pair offspring from polyandrous mothers from the same pair of social parents. **Figure S1**. The number of broods included in our analyses from 2000 to 2011. **Figure S2**. Pairwise differences between extra-pair offspring (EPO) and within-pair offspring from polyandrous mothers (WPOp) from the same social parent pair identity at four offspring life-history stages from collected data: (a) hatching rate, (b) nestling survival rate, as the proportion of hatched chicks survived to day 12 posthatching, which was close to the time for them to fledge, (c) recruitment rate, as the proportion of fledglings that produced at least one egg, and (d) the average number of fledglings that the recruited EPO or WPOp produced through their lifetime. **Figure S3**. Histograms of the observed lifetime reproductive output for extra-pair offspring (EPO), within-pair offspring from monogamous mothers (WPOm) and within-pair offspring from polygamous mothers (WPOp). **Table S1**. The normal distribution priors (for fixed effects) and inverse Wishart priors (for random effects) used in each MCMCglmm model. **Table S2**. Parameters in statistical models to test whether paternity group was associated with offspring fitness performance among extra-pair offspring (EPO), within-pair offspring from monogamous mothers (WPOm) and within-pair offspring from polyandrous mothers (WPOp). **Table S3**. Results from the binomial generalized linear mixed model, GLMM, with logit-link function, explaining variation in hatching success for all. **Table S4**. Parameters in statistical models to test whether paternity was associated with offspring fitness performance between within-pair offspring from polyandrous mothers (WPOp) from broods with EPO and WPOp from pure broods, using WPOp from pure broods as the baseline. **Table S5**. The normal distribution priors (for fixed effects) and inverse Wishart priors (for random effects) used in each MCMCglmm model used to compare the fitness performance of within-pair offspring from polyandrous mothers (WPOp) from mixed broods and WPOp from pure broods. **Table S6**. Sample sizes for the analyses at four subsequent life-history stages and composite fitness. **Table S7**. Sample sizes for the analyses at four subsequent life-history stages and composite fitness for the comparisons between within-pair offspring from polyandrous mothers (WPOp) from mixed broods and WPOp from pure broods. **Table S8**. Contrasts (Group 1 and Group 2) of the lifetime reproductive output between any two groups of offspring when considering the interaction between paternity and sex. **Table S9**. Results from the zero-inflated Poisson generalized linear mixed model (ZIP GLMM) using a combination of binomial error with logit-link function and Poisson error with log-link function. **Table S10**. Parameters in statistical models to test whether paternity was associated with offspring fitness performance between extra-pair offspring (EPO) and within-pair offspring from polyandrous mothers from the same pair of social parents (WPOp), with WPOp as the baseline. **Table S11**. Sample size for analyses at each life-history stage for paired tests for extra-pair offspring (EPO) and within-pair offspring from polyandrous mothers (WPOp). **Table S12**. Results from the generalized linear mixed models, GLMMs, explaining variation in hatching success, nestling survival, recruitment, and lifetime reproductive output, for paired tests between extra-pair offspring (EPO) and within-pair offspring from polyandrous mothers (WPOp) from the same social parent pair identity, with the WPOp as the baseline.
