# Supplementary material for: Costly Infidelity: Low Lifetime Fitness of Extra-Pair Offspring in a Passerine Bird
Source: Evolution. 2014 Jul 22;68(10):2873–84. doi: 10.1111/evo.12475 (PMC4303991; doi:10.1111/evo.12475)
Supplement: Supplementary file 1 — Supporting Information S1. Detailed methods for statistical analysis in the main text. Supporting Information S2. Models comparing extra-pair offspring and within-pair offspring from polyandrous mothers from the same pair of social parents. Figure S1. The number of broods included in our analyses from 2000 to 2011. Figure S2. Pairwise differences between extra-pair offspring (EPO) and within-pair offspring from polyandrous mothers (WPOp) from the same social parent pair identity at four offspring life-history stages from collected data: (a) hatching rate, (b) nestling survival rate, as the proportion of hatched chicks survived to day 12 posthatching, which was close to the time for them to fledge, (c) recruitment rate, as the proportion of fledglings that produced at least one egg, and (d) the average number of fledglings that the recruited EPO or WPOp produced through their lifetime. Figure S3. Histograms of the observed lifetime reproductive output for extra-pair offspring (EPO), within-pair offspring from monogamous mothers (WPOm) and within-pair offspring from polygamous mothers (WPOp). Table S1. The normal distribution priors (for fixed effects) and inverse Wishart priors (for random effects) used in each MCMCglmm model. Table S2. Parameters in statistical models to test whether paternity group was associated with offspring fitness performance among extra-pair offspring (EPO), within-pair offspring from monogamous mothers (WPOm) and within-pair offspring from polyandrous mothers (WPOp). Table S3. Results from the binomial generalized linear mixed model, GLMM, with logit-link function, explaining variation in hatching success for all. Table S4. Parameters in statistical models to test whether paternity was associated with offspring fitness performance between within-pair offspring from polyandrous mothers (WPOp) from broods with EPO and WPOp from pure broods, using WPOp from pure broods as the baseline. Table S5. The normal distribution priors (for fixed effects [file evo0068-2873-sd1.zip › CostlyInfidelity_SupportingInformation1.pdf]

Supporting Information 1.
Detailed methods for statistical analysis in the main text

We ran two sets of analyses, one to compare fitness performance among extra-pair offspring (EPO), within-pair offspring from monogamous mothers (WPOm), and within-pair offspring from polyandrous mothers (WPOp), and the other to compare between WPOp from mixed broods (broods with EPO) and WPOp from broods without EPO (pure broods). We ran each final model with three independent MCMC chains. The between-chain convergences among these three chains were examined by Gelman and Rubin's convergence diagnostic in R package, Coda (Gelman and Rubin 1992; Brooks and Gelman 1998; Plummer et al. 2006). We also calculated the autocorrelation for fixed and random effects, separately, to examine the within-chain independence between successive samples in the chain for the final model. For all models, we *z*-transformed clutch size and the first-laying day so that regression coefficients from GLMMs are readily interpretable (Schielzeth 2010).

Comparisons among EPO, WPOm and WPOp

Unlike some other studies, which compared EPO and WPOp within the same brood, we consider WPOp at the level of the social pair. This is because in some species, e.g. house sparrows (*Passer domesticus*), the same social pair often breeds together for more than once within and between breeding seasons (Nakagawa and Gillespie et al. 2007). During these breeding attempts, the genetic quality of the male and the genetic dissimilarity between the male and the female remain the same. Thus, if a female chooses a male according to his genetic quality or genetic dissimilarity, we expect her mating decision to be consistent between different broods within the same pair bond. For each model, we considered all potential main effects, e.g. paternity group and sex, and gradually added biologically meaningful interactions and quadratic effects, e.g., the interaction between sex and paternity group, because the effects of paternity might be sex-specific (Kokko 2001; Sardell et al. 2011; variables considered in each model were listed in

Table S2). We removed statistically nonsignificant fixed effects. We also removed random effects if their lower 95% CI was smaller than 0.001 if their removal increased the likelihood of a model converging. For each final model, we always kept the paternity group (EPO, WPOm, WPOp) as a fixed factor, as it was our variable of interest.

We used a binary GLMM (binomial error with logit-link function) to investigate whether paternity group was associated with hatching success. Paternity group and sex were set as fixed effects. As random effects, we included cohort (the year in which the focal individual was born) to account for annual variation, and biological brood identity (the brood that an egg was laid) to account for a common early environment (Kruuk and Hadfield 2007). However, although adding a cohort effect is biologically meaningful, this effect caused model instability, resulting in unrealistically wide 95% CIs for fixed effects. Therefore, we present results from the model without cohort in the main text, and report results from a model with cohort here in the supporting information (Table S3).

We investigated whether nestling survival was associated with paternity group using a binary GLMM. We included paternity group as a fixed effect, but we also included the first laying day as a fixed effect because it is known that the stage of the season can affect food availability and therefore nestling survival (Grant et al. 2005). We defined the first laying day as the date on which the first egg was laid in that particular brood as the Julian day, with 1 January as Julian day 1. We also included clutch size as a fixed effect, defined as the maximum number of eggs or chicks born in a brood, because clutch size is known to influence nestling survival (Murphy 2000). We included the growing-up brood identity (the brood in which a chick was raised) as a random effect. During the long-term study on Lundy Island, we have routinely cross-fostered chicks without changing clutch size since 2000, so that some chicks were not raised by their natal parents (Schroeder et al. 2011). To test for a potential bias arising from this cross-fostering, we split up our data into cross-fostered and non-cross-fostered clusters and analysed them separately. Both clusters showed qualitatively the same patterns of effects of paternity in preliminary analyses, so we combined both data sets for the analyses presented here.

We ran another binary GLMM to examine whether recruitment was associated with paternity group. We used paternity group as a fixed effect and cohort as a random effect. For each of the

binary GLMMs described above, we added an additive dispersion parameter, which is akin to the residual variance in linear mixed models, and fixed it to 1. This is because dispersion in binary models is unidentifiable or 0 (note that the model will not run with the dispersion parameter fixed to 0; see Nakagawa and Schielzeth 2010). Thus, we later rescaled the estimates of fixed effects to the values expected under additive dispersion of 0 (MCMCglmm course note; Hadfield 2012).

To investigate whether paternity group was associated with the lifetime reproductive output of recruited individuals, we used Poisson GLMMs (Poisson error with log-link function). We included paternity group and sex as fixed effects. The interaction between paternity group and sex was modelled to test whether the paternity group effect varied with sex (Sardell et al. 2012). We included the cohort, the social parent-pair identity and the growing-up brood identity as random effects. Because male and female lifetime reproductive output are interdependent, we ran three different models to control for this correlated structure, including (a) a model that accounted for the interaction between sex and paternity group, (b) a model with heterogeneous residuals for males and females, and (c) an analysis in which we divided males and females into two data sets and analysed them separately. Results from all three methods were similar to each other, so here we only present results from the model that included the interaction (a).

To estimate how composite fitness was associated with paternity group, we used a zero-inflated Poisson (ZIP) GLMM without intercept. ZIP is a method to model count data with an excess of zero counts using a combination of a binomial error with logit-link function and a Poisson error with log-link function (Hadfield 2010). ZIP has two predictors: one to predict the zero inflation and one to predict the Poisson counts (Zuur et al. 2012). We used the zero-inflated process to model how paternity group associated with the probability of each embryo reaching adulthood. Thus, a higher estimate indicates a lower probability of an embryo surviving. The Poisson component indicates the association between paternity group and the number of fledglings each survived adult produced during its lifetime (lifetime reproductive success, LRS; could be ≥ 0). We fitted paternity group and sex as fixed factors. To account for the correlation structure of male and female LRS, we included the interaction between paternity group and sex as a fixed effect (see above). We fitted cohort, biological brood identity and the social parent-pair identity

as random effects to account for environmental variation and correlated structure within the same brood, or the same pair of social parents (Kruuk and Hadfield 2007).

For models without an intercept, we conducted post-hoc (contrast) analyses at the latent scales to test for significance of differences between any two paternity groups (e.g. EPO, WPOm and WPOp). To allow biological interpretation, predictions of some fixed-effect estimates were calculated by back-transforming the estimates using the approximation method in Diggle et al. (2002).

Comparisons between WPOp from mixed broods and WPOp from pure broods

To disentangle potential direct costs and potential indirect benefits, we compared the fitness performance between WPOp from mixed broods and WPOp from pure broods. We extracted a subset of data with only WPOp, and assigned them into two groups, one with EPO in biological broods and another without EPO (using “no EPO” as the baseline). We used binary GLMMs with intercept to investigate whether having EPO in brood influenced (1) nestling survival and (2) recruitment, and we used Poisson GLMMs with intercepts to investigate whether having EPO in the brood influenced the lifetime reproductive output of recruited fledglings. Effects included and the combinations of inverse Wishart priors for each MCMCglmm model are listed in Table S4 and Table S5, respectively.

References

Brooks, S. P. and A. Gelman. 1998. General methods for monitoring convergence of iterative simulations. J. Comp. Graph. Stat. 7:434-455.

Diggle, P. J., P. Heagerty, K.-Y. Liang, and S. L. Zeger. 2002. Analysis of longitudinal data. Oxford University Press.

Gelman, A. and D. B. Rubin. 1992. Inference from iterative simulation using multiple sequences. Stat. Sci, 7:457-511.

Grant, T. A., T. L. Shaffer, E. M. Madden, and P. J. Pietz. 2005. Time-specific variation in passerine nest survival: New insights into old questions. Auk 122:661-672.

Hadfield, J. D. 2010. MCMC methods for multi-response generalized linear mixed models: The MCMCglmm R package. J. Stat. Softw. 33:1-22.

Kokko, H. 2001. Fisherian and "good genes" benefits of mate choice: How (not) to distinguish between them. Ecol. Lett. 4:322-326.

Kruuk, L. E. B. and J. D. Hadfield. 2007. How to separate genetic and environmental causes of similarity between relatives. J. Evol. Biol. 20:1890-1903.

Murphy, M. T. 2000. Evolution of clutch size in the eastern kingbird: Tests of alternative hypotheses. Ecol. Monogr. 70:1-20.

Nakagawa, S., D. O. S. Gillespie, B. J. Hatchwell, and T. Burke. 2007. Predictable males and unpredictable females: Sex difference in repeatability of parental care in a wild bird population. J. Evol. Biol. 20:1674-1681.

Nakagawa, S. and H. Schielzeth. 2010. Repeatability for Gaussian and non-Gaussian data: A practical guide for biologists. Biol. Rev. 85:935-956.

Plummer, M., N. Best, K. Cowles, and K. Vines. 2006. CODA: convergence diagnosis and output analysis for MCMC. R News 6:7–11.

Sardell, R. J., P. Arcese, L. F. Keller, and J. M. Reid. 2011. Sex-specific differential survival of extra-pair and within-pair offspring in song sparrows, *Melospiza melodia*. Proc. R. Soc. Lond. B 278:3251-3259.

Sardell, R. J., P. Arcese, L. F. Keller, and J. M. Reid. 2012. Are there indirect fitness benefits of female extra-pair reproduction? Lifetime reproductive success of within-pair and extra-pair offspring. Am. Nat. 179:779-793.

Schielzeth, H. 2010. Simple means to improve the interpretability of regression coefficients. Methods Ecol. Evol. 1:103-113.

Schroeder, J., I. R. Cleasby, S. Nakagawa, N. Ockendon, and T. Burke. 2011. No evidence for adverse effects on fitness of fitting passive integrated transponders (PITs) in wild house sparrows *Passer domesticus*. J. Avian Biol. 42:271-275.

Zuur, A. F., A. A. Savaliey, and E. N. Ieno. 2012. Zero inflated models and generalized linear mixed models with R. Highland Statistics Ltd, Newburgh, GB.
