# Supplementary material for: Costly Infidelity: Low Lifetime Fitness of Extra-Pair Offspring in a Passerine Bird
Source: Evolution. 2014 Jul 22;68(10):2873–84. doi: 10.1111/evo.12475 (PMC4303991; doi:10.1111/evo.12475)
Supplement: Supplementary file 1 — Supporting Information S1. Detailed methods for statistical analysis in the main text. Supporting Information S2. Models comparing extra-pair offspring and within-pair offspring from polyandrous mothers from the same pair of social parents. Figure S1. The number of broods included in our analyses from 2000 to 2011. Figure S2. Pairwise differences between extra-pair offspring (EPO) and within-pair offspring from polyandrous mothers (WPOp) from the same social parent pair identity at four offspring life-history stages from collected data: (a) hatching rate, (b) nestling survival rate, as the proportion of hatched chicks survived to day 12 posthatching, which was close to the time for them to fledge, (c) recruitment rate, as the proportion of fledglings that produced at least one egg, and (d) the average number of fledglings that the recruited EPO or WPOp produced through their lifetime. Figure S3. Histograms of the observed lifetime reproductive output for extra-pair offspring (EPO), within-pair offspring from monogamous mothers (WPOm) and within-pair offspring from polygamous mothers (WPOp). Table S1. The normal distribution priors (for fixed effects) and inverse Wishart priors (for random effects) used in each MCMCglmm model. Table S2. Parameters in statistical models to test whether paternity group was associated with offspring fitness performance among extra-pair offspring (EPO), within-pair offspring from monogamous mothers (WPOm) and within-pair offspring from polyandrous mothers (WPOp). Table S3. Results from the binomial generalized linear mixed model, GLMM, with logit-link function, explaining variation in hatching success for all. Table S4. Parameters in statistical models to test whether paternity was associated with offspring fitness performance between within-pair offspring from polyandrous mothers (WPOp) from broods with EPO and WPOp from pure broods, using WPOp from pure broods as the baseline. Table S5. The normal distribution priors (for fixed effects [file evo0068-2873-sd1.zip › CostlyInfidelity_SupportingInformation2.pdf]

Supporting Information 2:

Models comparing extra-pair offspring and within-pair offspring from polyandrous mothers from the same pair of social parents

Introduction and Methods

In the models in the main text, we estimated the paternity effect across offspring from different pairs of social parents, including extra-pair offspring (EPO), within-pair offspring from monogamous mothers (WPOm) and within-pair offspring from polygamous mothers (WPOp). To account for correlated structures in the datasets (e.g., multiple chicks from the same mother), we included factors such as dam identity, genetic sire identity and the social parent pair identity as random effects in generalized linear mixed models (GLMMs). However, assuming paternity effects influence offspring fitness within the same pair of social parents, such effect might not be detected between different pair of social parents under certain situations (Schielzeth and Forstmeier 2009). Our models in the main text do not account for this effect because such an effect can only be modeled with a subset of the data, which includes only EPO and WPOp from social parents who had both EPO and WPOp. Therefore, we extracted a subset of data from the main datasets for this purpose. Importantly, to estimate paternity effects within social parents who had both EPO and WPOp, we employed random-slope GLMMs (note that the models in the main text are random-intercept mixed-effect models; Schielzeth and Forstmeier 2009).

We used binary GLMMs (binomial error with logit-link function) with intercept and random slopes to investigate whether paternity influenced (1) hatching success, (2) nestling survival, and (3) recruitment, and we used Poisson GLMMs (Poisson error with log-link function) with random slopes to investigate whether paternity effect influenced the lifetime reproductive output of recruited fledglings. See the main text for details of the definition of fitness components and statistical analyses. These models that compared fitness components between EPO and WPOp from the same pair of social parents were referred to as paired tests hereafter. For each model for the paired tests, we started by considering all potential factors at the beginning and gradually removed factors that did not significantly influence offspring fitness performance at each stage.

We listed the variables included in each model in Table S10 and the combinations of inverse Wishart priors in Table S1.

Results and Discussion

In total, we included 2,036 offspring from 191 pairs of social parents in the paired tests; among them, 504 were EPO and 1,532 were WPOp. Because we only included individuals that succeeded in the previous life-history stage in the analysis of the following life-history stage, the sample sizes reduced along the stages (Table S11).

Paternity did not influence the probability of each egg hatching (Table S12, Figure S2a). A male offspring was estimated to have a 0.8% higher chance of hatching than a female offspring from the same pair of social parents. This difference was small but statistically significant. The probability of each hatched nestling surviving to the day 12 post-hatching was not influenced by paternity (Table S12, Figure S2b). However, a male nestling was estimated to have a 9.4% higher chance of surviving than a female nestling. This difference was statistically significant. An increase in one standard deviation in clutch size (which was 0.9 eggs) was estimated to incur a 4.4% reduction in the probability of nestling survival. Also, an increase in one standard deviation in the first egg-laying date (which was 31.1 days) was estimated to lead to a 7.5% increase in the chance of nestling survival. Both the clutch size and first egg-laying date effects were statistically significant (Table S12).

Paternity could not explain the variation in the probability of a fledgling recruiting into the breeding population (Table S12, Figure S2c). However, a male fledgling was estimated to have a 7.0% higher chance of being recruited than a female fledgling; the difference was statistically significant. The number of fledglings produced by one individual through its lifetime was not influenced by paternity, sex, or the interaction of paternity and sex (Table S12, Figure S2d). However, on average, a male EPO was estimated to produce one fledgling through its lifetime, which was significantly lower than female WPOp (four fledglings).

Through these four life-history stages, paternity had a negative but nonsignificant effect on offspring fitness (Table S12). Thus, in general, the results of paired tests between EPO and

WPOp from the same pair of social-pair parents were consistent with those in the comparisons among EPO, WPOp and WPOm. The only difference was that the 95% credible intervals (95% CIs) of the paternity effect in paired tests were wider than those for comparisons among EPO, WPOp and WPOm, and these wider 95% CIs were due to lower statistical power in the paired test for lifetime reproductive output than the corresponding model in the main text. The power reduced because that for the paired tests, we could only include social-pair parents who produced both EPO and WPOp at that life-history stage. Nevertheless, the results remained in the same direction as in comparisons among all three groups of offspring, which indicated no support for the good genes or genetic compatibility hypotheses, as discussed in the main text.

References

Schielzeth, H. and W. Forstmeier. 2009. Conclusions beyond support: Overconfident estimates in mixed models. Behav. Ecol. 20:416-420.
