# Supplementary material for: Costly Infidelity: Low Lifetime Fitness of Extra-Pair Offspring in a Passerine Bird
Source: Evolution. 2014 Jul 22;68(10):2873–84. doi: 10.1111/evo.12475 (PMC4303991; doi:10.1111/evo.12475)
Supplement: Supplementary file 1 — Supporting Information S1. Detailed methods for statistical analysis in the main text. Supporting Information S2. Models comparing extra-pair offspring and within-pair offspring from polyandrous mothers from the same pair of social parents. Figure S1. The number of broods included in our analyses from 2000 to 2011. Figure S2. Pairwise differences between extra-pair offspring (EPO) and within-pair offspring from polyandrous mothers (WPOp) from the same social parent pair identity at four offspring life-history stages from collected data: (a) hatching rate, (b) nestling survival rate, as the proportion of hatched chicks survived to day 12 posthatching, which was close to the time for them to fledge, (c) recruitment rate, as the proportion of fledglings that produced at least one egg, and (d) the average number of fledglings that the recruited EPO or WPOp produced through their lifetime. Figure S3. Histograms of the observed lifetime reproductive output for extra-pair offspring (EPO), within-pair offspring from monogamous mothers (WPOm) and within-pair offspring from polygamous mothers (WPOp). Table S1. The normal distribution priors (for fixed effects) and inverse Wishart priors (for random effects) used in each MCMCglmm model. Table S2. Parameters in statistical models to test whether paternity group was associated with offspring fitness performance among extra-pair offspring (EPO), within-pair offspring from monogamous mothers (WPOm) and within-pair offspring from polyandrous mothers (WPOp). Table S3. Results from the binomial generalized linear mixed model, GLMM, with logit-link function, explaining variation in hatching success for all. Table S4. Parameters in statistical models to test whether paternity was associated with offspring fitness performance between within-pair offspring from polyandrous mothers (WPOp) from broods with EPO and WPOp from pure broods, using WPOp from pure broods as the baseline. Table S5. The normal distribution priors (for fixed effects [file evo0068-2873-sd1.zip › CostlyInfidelity_SupportingInformation3_TablesAndFigures.pdf]

Supporting information 3: Tables and figures

Table S1. The normal distribution priors (for fixed effects) and inverse Wishart priors (for random effects) used in each MCMCglmm model. Here, EPO indicates extra-pair offspring, WPOp indicates within-pair offspring from polyandrous mothers, and WPOm indicates within-pair offspring from monogamous mothers. The inverse Wishart prior has two parameters: *V* defined variance, and *nu* defined the degree of belief in *V*. B specified the structure of fixed effects, and also included the expected value (*mu*), whereas R specified the structure of residual and G specified the structure of random effects. *p.var* was defined as the variance of the response variable in that particular model. Varying *V* values were given based on preliminary results. For the R structure in binary GLMMs, including models for hatching success, nestling survival and recruitment, we fixed the additive dispersion parameter to 1 (i.e., *fix=1*).

Model	Priors
**Among EPO, WPOp and WPOm**	
Hatching success (did not include cohort effect)	B=list(*mu*=rep(0,4), *V*=diag(4)*(1+*pi*^2/3)),<br>R=list(*V*=1, fix=1),<br>G=list(G1=list(*V*=10, *nu*=1e-5))
Hatching success (consider cohort as a random effect)	B=list(*mu*=rep(0,4), *V*=diag(4)*(1+*pi*^2/3)),<br>R=list(*V*=1, fix=1),<br>G=list(G1=list(*V*=25, *nu*=1e-5), G2=list(*V*=7.4, *nu*=1e-5))
Nestling survival	B=list(*mu*=rep(0,5), *V*=diag(5)*(1+*pi*^2/3)),<br>R=list(*V*=1, fix=1),<br>G=list(G1=list(*V*=9, *nu*=1e-4))
Recruitment	B=list(*mu*=rep(0,3), *V*=diag(3)*(1+*pi*^2/3)),<br>R=list(*V*=1, fix=1),<br>G=list(G1=list(*V*=0.45, *nu*=1e-4))
Lifetime reproductive output	R=list(*V*=0.58, *nu*=1e-5)<br>G=list(G1=list(*V*=0.29, *nu*=1e-3), G2=list(*V*=0.05, *nu*=1e-3), G3=list(*V*=0.07, *nu*=1e-3))
Composite fitness	R=list(*V*=diag(2), *nu*=0.05,fix=2),<br>G=list(G1=list(*V*=diag(2), *nu*=2), G2=list(*V*=diag(2), *nu*=2), G3=list(*V*=diag(2), *nu*=2))
**Paired tests between EPO and WPOp from the same pair of social parents**	
Hatching success	B=list(*mu*=rep(0,5), *V*=diag(5)*(1+*pi*^2/3)),<br>R=list(*V*=1, fix=1),<br>G=list(G1=list(*V*=28, *nu*=1e-5), G2=list(*V*=7, *nu*=1e-5))
Nestling survival	B=list(*mu*=rep(0,5), *V*=diag(5)*(1+*pi*^2/3))<br>R=list(*V*=1, fix=1),<br>G=list(G1=list(*V*=diag(2), *nu* = 1e-4), G2=list(*V*=*p.var*/3, *nu*=1e-4),<br>G3=list(*V*=*p.var*/3, *nu*=1e-4))
Recruitment	B=list(*mu*=rep(0,3), *V*=diag(3)*(1+*pi*^2/3)),<br>R=list(*V*=1, fix=1),<br>G=list(G1=list(*V*=diag(2), *nu*=1e-4), G2=list(*V*=*p.var*/2, *nu*=1e-5))
Lifetime reproductive output	R=list(*V*=168, *nu*=1e-5),<br>G=list(G1=list(*V*=diag(2), *nu*=1e-4), G2=list(*V*=0.61, *nu*=1e-3), G3=list(*V*=0.1, *nu*=1e-3))

Table S2. Parameters in statistical models to test whether paternity group was associated with offspring fitness performance among extra-pair offspring (EPO), within-pair offspring from monogamous mothers (WPOm) and within-pair offspring from polyandrous mothers (WPOp). Separate models were run for the four different fitness components: hatching success, nestling survival, recruitment and lifetime reproductive output. In this table, '++' indicates effects included in the final model while 'v' indicates the effects that were considered in the sequential model selection, but not included in the final model. Lifetime reproductive output was defined as the number of fledglings that each individual produced through their lifetime.

Model parameters	Hatching	Nestling Survival	Recruitment	Lifetime reproductive output
Fixed Effects				
Paternity group	++	++	++	++
Sex	++	v	v	++
z-transformed clutch size	v	++	v	
z-transformed first-laying Day	v	++		
(z-transformed clutch size)^2	v	v		
(z-transformed first-laying Day)^2	v	v		
Paternity group * Sex	v	v		++
Random Effects				
Cohort	v	v	++	++
Biological brood§	++	v	v	v
Social parent pair	v	v	v	++
Dam	v	v	v	v
Dam age	v	v	v	v
Genetic sire	v	v	v	v
Growing-up brood§		++	v	++
Parameters in MCMC process				
Burn-in length	5,000,000	5,000,000	15,000,000	5,000,000
Iteration	5,000,000	5,000,000	10,000,000	5,000,000
Number of posterior samples	10,000	10,000	10,000	10,000

§: Because we routinely cross-fostered chicks without changing the clutch size during the long term-study on Lundy, for some chicks the growing-up brood identity was different from their biological (original) brood identity. Both brood identities might have an influence on nestling survival onwards, so we considered both of them as random effects in the models.

Table S3. Results from the binomial generalized linear mixed model, GLMM, with logit-link function, explaining variation in hatching success for all. This model is different from the model from which we present the results in the main text, because the model here included the cohort in which each egg was laid as a random effect. Rescaled posterior means and 95% credible intervals (95% CIs) under the additive dispersion of 0 are presented. Here, 'Sex' indicates the difference between male and female, with female as the baseline. EPO: extra-pair offspring, WPOp: within-pair offspring from polyandrous mothers, and WPOm: within-pair offspring from monogamous mothers.

Model	Hatching	
Estimate	Mean	95% CI
**Fixed Effects**		
EPO	1.75	-0.65 to 3.78
WPOp	1.96	-0.29 to 4.09
WPOm	1.50	-0.75 to 3.69
Sex	0.50	0.17 to 0.81
**Random Effects**		
Cohort	47.55	2.44 to 140.95
Biological brood	5.53	3.74 to 7.36

Table S4. Parameters in statistical models to test whether paternity was associated with offspring fitness performance between within-pair offspring from polyandrous mothers (WPOp) from broods with EPO and WPOp from pure broods, using WPOp from pure broods as the baseline. Separate models were run for the three fitness components: nestling survival, recruitment and lifetime reproductive output. In this table, '++' indicates effects included in the final model, 'v' indicates the effects that were considered in the sequential model selection, but not included in the final model. Lifetime reproductive output was calculated by the number of fledglings that each individual produced through its lifetime.

Model Parameter	Nestling Survival	Recruitment	Lifetime reproductive output
Fixed Effects			
EPO in brood	++	++	++
z-transformed clutch size	++		
z-transformed first-laying Day	++		
Random Effects			
Cohort	++	++	++
Growing-up brood	++	++	++
Social parent pair	++	++	++
Dam identification	++	++	++
Parameters in MCMC process			
Burn-in length	5,000,000	5,000,000	5,000,000
Iteration	10,000,000	10,000,000	10,000,000
Number of posterior samples	10,000	10,000	10,000

Table S5. The normal distribution priors (for fixed effects) and inverse Wishart priors (for random effects) used in each MCMCglmm model used to compare the fitness performance of within-pair offspring from polyandrous mothers (WPOp) from mixed broods and WPOp from pure broods. Inverse Wishart prior has two parameters: *V* defined variance, and *nu* defined the degree of belief in *V*. B specified the structure of fixed effects, and also included the expected value (*mu*), whereas R specified the structure of residual and G specified the structure of random effects. *p.var* was defined as the variance of the response variable in that particular model. Varying *V* values were given based on preliminary results. For the R structure in binary GLMMs, including models for hatching success, nestling survival and recruitment, we fixed the additive dispersion parameter to 1 (i.e., *fix=1*).

Model	Priors
Nestling survival	B=list(*mu*=rep(0,4), *V*=diag(4)*(1+*pi*^2/3)),<br>R=list(*V*=1, fix=1),<br>G=list(G=list(G1=list(V=1, nu=1e-4), G2=list(V=1,nu=1e-4),G3=list(V=1, nu=1e-4), G4=list(V=1,nu=1e-4))
Recruitment	B=list(*mu*=rep(0,2), *V*=diag(2)*(1+*pi*^2/3)),<br>R=list(*V*=1, fix=1),<br>G=list(G1=list(V=1,nu=1e-4), G2=list(V=1,nu=1e-4),G3=list(V=1,nu=1e-4), G5=list(V=1,nu=1e-4))
Lifetime reproductive output	R=list(*V*=1, *nu*=1e-5)<br>G=list(G1=list(V=1,nu=1e-3), G2=list(V=1, nu=1e-3),G3=list(V=1,nu=1e-3), G4=list(V=1,nu=1e-3))

Table S6. Sample sizes for the analyses at four subsequent life-history stages and composite fitness. Only individuals that successfully survived in the previous stage were included in the analysis of the subsequent life-history stage. For the analysis of lifetime reproductive output, we included only recruited fledglings that died before February 2012. For the analysis of composite fitness, we included all individuals that died before February 2012. EPO: extra-pair offspring. WPOm: with-pair offspring from monogamous mothers. WPOp: within-pair offspring from polyandrous mothers.

Model	Hatch	Nestling survival	Recruitment	Lifetime reproductive output	Composite fitness
Total offspring	3285	2913	1522	302	2902
Biological brood	965	912	607	229	854
Social parent pairs	436	419	327	157	382
**EPO**	**591**	**523**	**239**	**34**	**484**
Female	309	273	119	15	244
Male	282	250	120	19	240
**WPOp**	**1590**	**1414**	**729**	**134**	**1440**
Female	791	696	361	62	716
Male	799	718	368	72	724
**WPOm**	**1104**	**976**	**554**	**134**	**978**
Female	578	492	285	70	518
Male	526	484	269	64	460

Table S7. Sample sizes for the analyses at four subsequent life-history stages and composite fitness for the comparisons between within-pair offspring from polyandrous mothers (WPOp) from mixed broods and WPOp from pure broods. Only individuals that successfully survived in the previous stage were included in the analysis of the subsequent life-history stage. For the analysis of lifetime reproductive output, we included only recruited fledglings that died before February 2012. For the analysis of composite fitness, we included all individuals that died before February 2012.

Model	Nestling survival	Recruitment	Lifetime reproductive output
Total offspring	1414	729	134
Biological brood	532	342	108
Social parent pair	199	161	69
WPOp with EPO in broods	607	221	49
WPOp without EPO in broods	807	508	85

Table S8. Contrasts (Group 1 – Group 2) of the lifetime reproductive output between any two groups of offspring when considering the interaction between paternity and sex. The estimates of posterior mean and their 95% credible interval (95%CI) are presented. Comparisons in bold are statistically significant. EPO: extra-pair offspring. WPOm: within-pair offspring from monogamous mothers. WPOp: within-pair offspring from polygamous mothers.

Comparisons		Estimation	
Group 1	Group 2	Mean	95% CI
♀ EPO	♀ WPOp	0.08	-0.48 to 0.65
♀ EPO	♀ WPOm	-0.06	-0.63 to 0.50
♀ **EPO**	♂ **EPO**	**0.82**	**0.09 to 1.53**
♀ EPO	♂ WPOp	0.13	-0.42 to 0.69
♀ EPO	♂ WPOm	0.09	-0.48 to 0.67
♀ WPOp	♀ WPOm	-0.06	-0.50 to 0.21
♀ **WPOp**	♂ **EPO**	**0.74**	**0.16 to 1.30**
♀ WPOp	♂ WPOp	0.05	-0.29 to 0.39
♀ WPOp	♂ WPOm	0.01	-0.37 to 0.37
♀ **WPOm**	♂ **EPO**	**0.88**	**0.29 to 1.43**
♀ WPOm	♂ WPOp	0.19	-0.16 to 0.55
♀ WPOm	♂ WPOm	0.15	-0.17 to 0.51
♂ **EPO**	♂ **WPOp**	**-0.69**	**-1.25 to -0.12**
♂ **EPO**	♂ **WPOm**	**-0.73**	**-1.33 to -0.17**
♂ WPOp	♂ WPOm	-0.04	-0.39 to 0.33

Table S9. Results from the zero-inflated Poisson generalized linear mixed model (ZIP GLMM) using a combination of binomial error with logit-link function and Poisson error with log-link function. This model explains variation in composite fitness, defined as the number of fledglings produced through its lifetime for each embryo as extra-pair offspring (EPO), within-pair offspring from monogamous mothers (WPOm) or within-pair offspring from polygamous mothers (WPOp). The zero-inflation process modelled the probability of each individual failing to survive from an embryo to adulthood. The Poisson process modelled, for each survived adult, the total number of fledglings produced in an individual's lifetime (≥0). Posterior means and 95% credible intervals (95% CIs) are presented.

Estimate			Mean	95% CI
**Fixed effects**				
	Zero-inflated	EPO	3.63	2.48 to 4.92
		WPOp	2.95	1.97 to 4.02
		WPOm	2.61	1.58 to 3.62
		EPO : Sex	-0.59	-1.80 to 0.50
		WPOp : Sex	-0.38	-1.12 to 0.31
	Poisson	EPO	1.69	1.03 to 2.35
		WPOp	1.65	1.15 to 2.17
		WPOm	1.60	1.07 to 2.07
		EPO : Sex	-0.73	-1.61 to 0.20
		WPOp : Sex	-0.16	-0.77 to 0.45
		WPOm : Sex	-0.25	-0.80 to 0.32
**Random effects**				
	Zero-inflated	Cohort	2.14	0.57 to 4.44
		Biological brood	0.72	0.19 to 1.32
		Social parent pair	0.49	0.16 to 0.88
	Poisson	Cohort	0.45	0.13 to 0.91
		Biological brood	0.19	0.09 to 0.29
		Social parent pair	0.19	0.09 to 0.29
**Dispersion**				
	Zero-inflated		-	-
	Poisson		0.22	0.13 to 0.34

Table S10. Parameters in statistical models to test whether paternity was associated with offspring fitness performance between extra-pair offspring (EPO) and within-pair offspring from polyandrous mothers from the same pair of social parents (WPOp), with WPOp as the baseline. Separate models were run for the four fitness components: hatching success, nestling survival, recruitment and lifetime reproductive output. In this table, '++' indicates effects included in the final model, 'v' indicates the effects that were considered in the sequential model selection, but not included in the final model. Lifetime reproductive output was calculated by the number of fledglings that each individual produced through its lifetime.

Model Parameter	Hatching	Nestling Survival	Recruitment	Lifetime reproductive output
Fixed Effects				
Paternity	++	++	++	++
Sex	++	++	++	++
z-transformed clutch size	v	++		
z-transformed first-laying Day	v	++		
(z-transformed clutch size)^2	v	v		
(z-transformed first-laying Day)^2		v		
Paternity group * Sex	v	v	V	++
Random Effects				
Paternity :				
Social parent pair	++	++	++	++
Cohort	++	++	++	++
Biological brood§	++	v		
Growing-up brood§		++		++
Parameters in MCMC process				
Burn-in length	10,000,000	6,000,000	10,000,000	10,000,000
Iteration	5,000,000	4,000,000	4,000,000	5,000,000
Number of posterior samples	10,000	10,000	10,000	10,000

§: Because we routinely cross-fostered chicks without changing the clutch size during the long term-study on Lundy, for some chicks the growing-up brood identity was different from their biological (original) brood identity. Both brood identities might have an influence on nestling survival onwards, so we considered both of them as random effects in the models.

Table S11. Sample size for analyses at each life-history stage for paired tests for extra-pair offspring (EPO) and within-pair offspring from polyandrous mothers (WPOp). Nestling survival was defined as whether a hatched chick survived to day 12 post-hatching, which was close to the time for it to fledge. Lifetime reproductive output was calculated as the number of fledglings that each EPO or WPOp produced through its lifetime.

Model	Hatch	Nestling survival	Recruitment	Lifetime reproductive output
Total offspring	2036	1734	714	63
Biological brood	569	520	271	43
Social parent pair	191	173	107	20
**EPO**	**504**	**438**	**197**	**23**
Female	259	224	95	11
Male	245	214	102	12
**WPOp**	**1532**	**1296**	**517**	**40**
Female	757	635	246	17
Male	775	661	271	23

Table S12. Results from the generalized linear mixed models, GLMMs, explaining variation in hatching success, nestling survival, recruitment and lifetime reproductive output, for paired tests between extra-pair offspring (EPO) and within-pair offspring from polyandrous mothers (WPOp) from the same social parent pair identity, with the WPOp as the baseline. Here, 'Sex' indicates the difference between male and female, with female as the baseline. Posterior means and 95% credible intervals (95% CIs) are presented

Model	Hatching		Nestling survival		Recruitment		Lifetime reproductive output	
Estimate	Mean	95% CI	Mean	95% CI	Mean	95% CI	Mean	95% CI
**Fixed Effects**								
(Intercept)	3.97	2.43 to 5.68	0.94	0.15 to 1.73	-1.55	-2.25 to -0.92	1.50	0.51 to 2.45
Paternity	-0.01	-0.58 to 0.57	-0.17	-0.52 to 0.20	-0.29	-0.74 to 0.13	-0.32	-1.57 to 0.86
Sex	0.39	0.01 to 0.75	0.53	0.25 to 0.84	0.42	0.04 to 0.80	-0.60	-1.55 to 0.33
Paternity : Sex	-	-	-	-	-	-	-0.61	-2.22 to 1.11
First-laying Day	-	-	0.41	0.17 to 0.66	-	-	-	-
Clutch Size	-	-	-0.21	-0.42 to 0.01	-	-	-	-
**Random Effects**								
Cohort	8.14	0.45 to 23.88	1.48	0.31 to 3.27	0.86	0.12 to 2.01	0.70	0 to 2.29
Biological brood§	3.10	1.67 to 4.74	-	-	-	-	-	-
Growing-up Brood§	-	-	4.71	3.19 to 6.30	-	-	0.07	0 to 0.34
**Dispersion**	-	-	-	-	-	-	1.66	0.67 to 2.90
**Random Slope Effect**								
(Intercept): social parent pair	0.70	0 to 1.55	0.60	0 to 1.16	0.07	0 to 0.32	-	-
Paternity : social parent pair	0.88	0 to 3.49	0.11	0 to 0.61	0.05	0 to 0.28	-	-

§: Because we routinely cross-fostered chicks without changing the clutch size during the long term-study on Lundy, for some chicks the growing-up brood identity was different from their biological (original) brood identity.

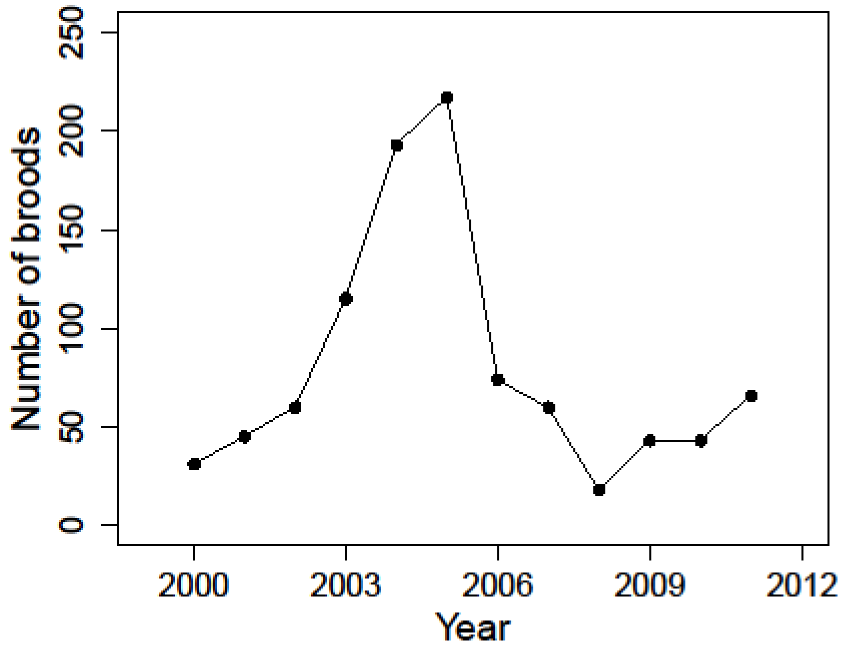

Figure S1. The number of broods included in our analyses from 2000 to 2011.

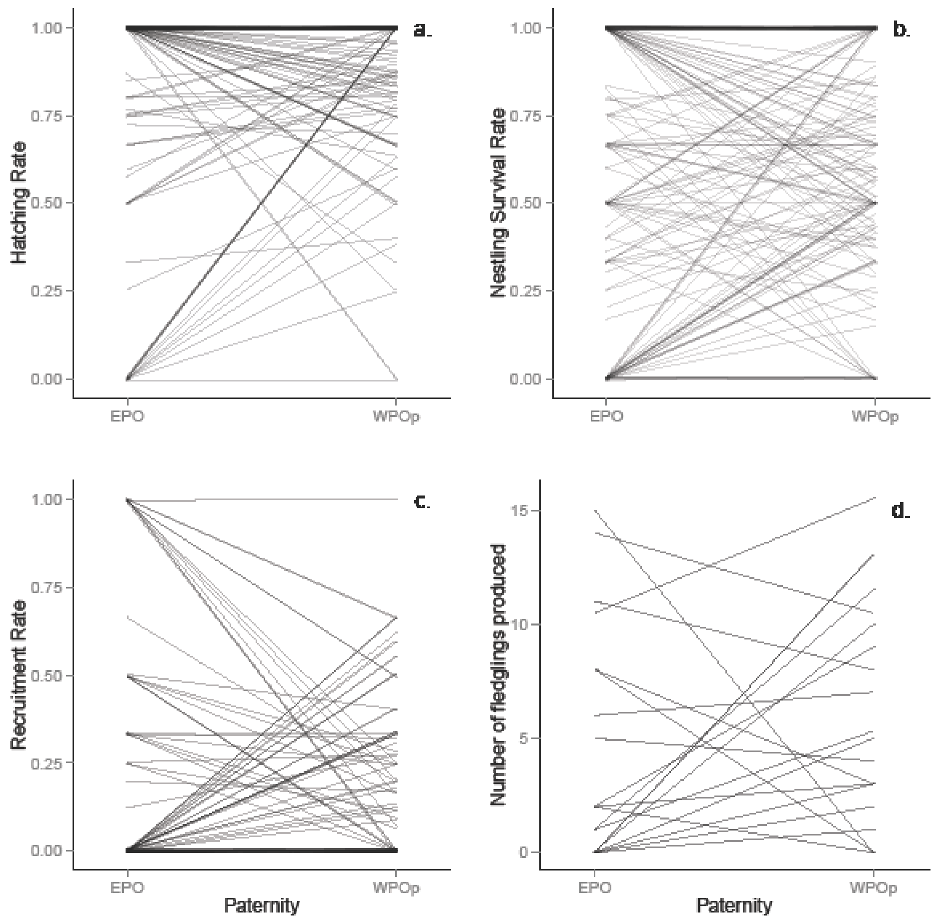

Figure S2. Pairwise differences between extra-pair offspring (EPO) and within-pair offspring from polyandrous mothers (WPOp) from the same social parent pair identity at four offspring life-history stages from collected data: a) hatching rate, b) nestling survival rate, as the proportion of hatched chicks survived to day 12 post-hatching, which was close to the time for them to fledge, c) recruitment rate, as the proportion of fledglings that produced at least one egg, and d) the average number of fledglings that the recruited EPO or WPOp produced through their lifetime. Each gray line indicates a pair of EPO and WPOp from one pair of social parents. The degree of gray in the lines indicates the number of pairs of EPO and WPOp, with darker lines indicating a larger number of EPO–WPOp pairs.

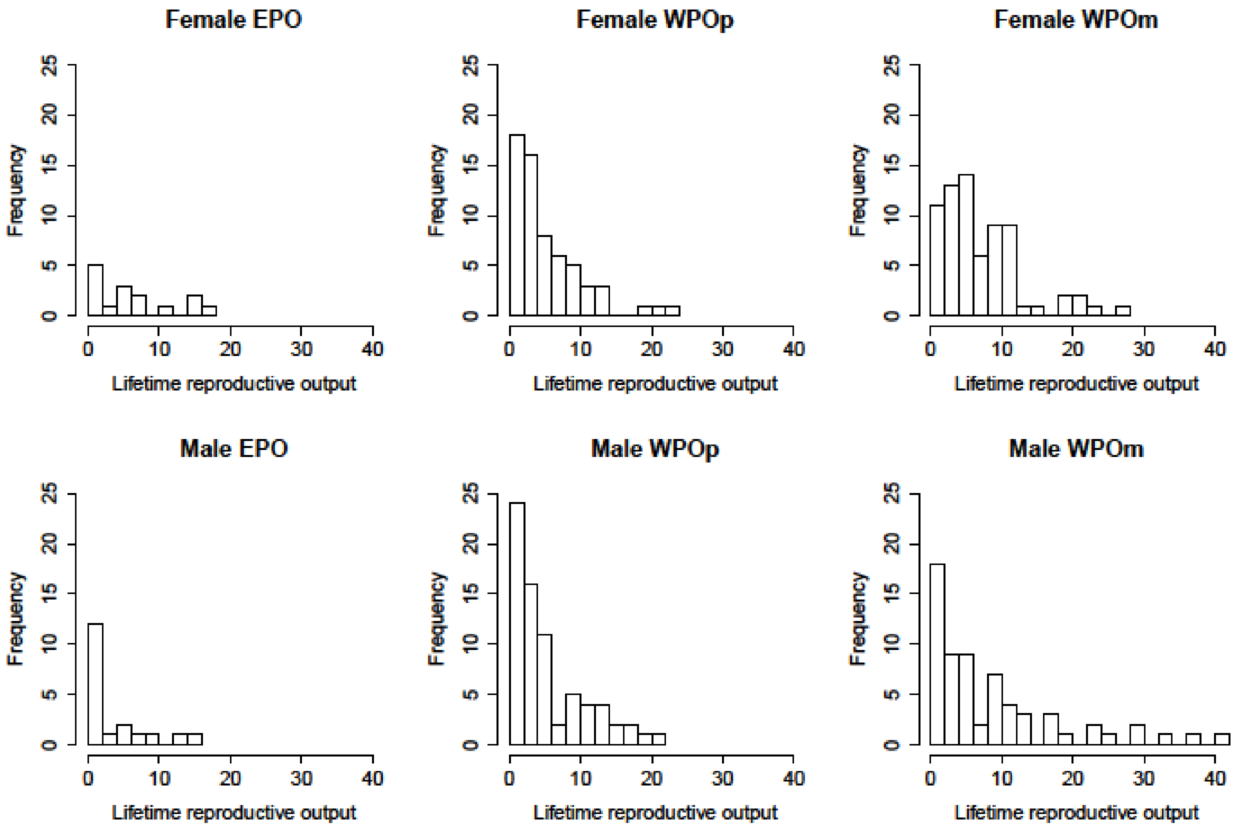

Figure S3. Histograms of the observed lifetime reproductive output for extra-pair offspring (EPO), within-pair offspring from monogamous mothers (WPOm) and within-pair offspring from polygamous mothers (WPOp). Lifetime reproductive output is defined as the number of fledglings produced by an individual through its lifetime.
